# Liking versus Complexity: Decomposing the Inverted U-curve

**DOI:** 10.3389/fnhum.2016.00112

**Published:** 2016-03-18

**Authors:** Yağmur Güçlütürk, Richard H. A. H. Jacobs, Rob van Lier

**Affiliations:** Donders Institute for Brain, Cognition and Behaviour, Radboud UniversityNijmegen, Netherlands

**Keywords:** liking, complexity, individual differences, clustering analysis, experimental aesthetics

## Abstract

The relationship between liking and stimulus complexity is commonly reported to follow an inverted U-curve. However, large individual differences among complexity preferences of participants have frequently been observed since the earliest studies on the topic. The common use of across-participant analysis methods that ignore these large individual differences in aesthetic preferences gives an impression of high agreement between individuals. In this study, we collected ratings of liking and perceived complexity from 30 participants for a set of digitally generated grayscale images. In addition, we calculated an objective measure of complexity for each image. Our results reveal that the inverted U-curve relationship between liking and stimulus complexity comes about as the combination of different individual liking functions. Specifically, after automatically clustering the participants based on their liking ratings, we determined that one group of participants in our sample had increasingly lower liking ratings for increasingly more complex stimuli, while a second group of participants had increasingly higher liking ratings for increasingly more complex stimuli. Based on our findings, we call for a focus on the individual differences in aesthetic preferences, adoption of alternative analysis methods that would account for these differences and a re-evaluation of established rules of human aesthetic preferences.

## Introduction

The relationship between the complexity of a stimulus and its perceived beauty has been a topic of great interest with influential studies since the earlier experimental investigations of aesthetics. For instance, Berlyne showed that complexity is a dominant determinant of interestingness and pleasingness of a stimulus (Berlyne, 1963; Berlyne et al., 1968). Berlyne (1971) suggested that the relationship between complexity and pleasingness could be explained by an inverted U-curve, where the stimuli with intermediate levels of complexity are the most preferable ones. This concept of an optimal amount of stimulus complexity has been supported by numerous studies that found an inverted U-curve when characterizing aesthetic preference as a function of complexity (Vitz, 1966; Berlyne, 1971; Saklofske, 1975; Farley and Weinstock, 1980; Imamoglu, 2000).

Although, Berlyne’s theory has been influential in the field of experimental aesthetics (Silvia, 2005), Nadal et al. (2010) point out some discrepancies among the results of previous studies investigating the relationship between liking and complexity. Nadal et al. (2010) illustrate that studies utilizing a systematic manipulation of the degree of stimulus complexity resulted not always in an inverted U-shaped characterization of aesthetic preference as a function of complexity, but sometimes increasing, decreasing or U-shaped characterizations of the relationship. Nadal et al. (2010) suggested that the results vary since different studies manipulated different complexity dimensions, and different complexity dimensions have different relationships with aesthetic preference. Although, we agree that the various complexity dimensions utilized in these studies could have a major effect on the divergence of the results, an additional factor contributing to this divergence could be the individual differences in aesthetic preferences of the participants. Large individual differences among complexity preferences of participants have been frequently observed even on a single study level (Vitz, 1966; Crosson and Robertson-Tchabo, 1983; Aks and Sprott, 1996; Jacobsen and Höfel, 2002). For instance, Jacobsen and Höfel (2002) found that, while the group-level analysis indicated a preference for higher levels of complexity, their sample of participants also included individuals who displayed exactly the opposite complexity preference patterns. These examples make it clear that it is desirable to look more carefully at individual differences. Individual differences in the experimental aesthetics literature are of course not limited to preference differences in complexity. More recently, a number of studies explored the relationship between characteristics of individuals (e.g., age, gender, educational background, and personality traits) and various types of aesthetic preferences such as preference for paintings of various artistic styles (Chamorro-Premuzic et al., 2009, 2010; Cleridou and Furnham, 2014), preference for architecture and music of various artistic styles (Cleridou and Furnham, 2014), preference for rectangles with various side length ratios (McManus et al., 2010), strength of musical aesthetic experiences (Nusbaum and Silvia, 2011) and harmony preference (Palmer and Griscom, 2013). The results of these studies were not entirely consistent with each other, such that for example, while Chamorro-Premuzic et al. (2009, 2010), and Cleridou and Furnham (2014) found correlations between various personality traits and aesthetic preferences, McManus et al. (2010), and Palmer and Griscom (2013) did not. Such inconsistencies between the results of aesthetic studies further emphasize the need for systematically investigating these preference differences.

Besides studying the preference differences on an individual level, we suggest that identifying subgroups of participants with similar preferences and then characterizing these subgroups can result in much progress in experimental aesthetics. To embark on such a task we suggest taking an exploratory approach and using clustering analysis. Since their initial use in psychology by Zubin (1938), clustering analysis methods have been embraced as a means of connecting the study of individuals (idiographic approach) and the study of cohorts of individuals (nomothetic approach), for example in health psychology, a field in which gaps between theory and individual cases are common (Clatworthy et al., 2005). Similarly, the differences between the nomothetic and idiographic approaches have been a concern in the study of aesthetics (Berlyne, 1977; Jacobsen and Höfel, 2002; Jacobsen, 2004; Mallon et al., 2014). Utilization of a clustering approach in experimental aesthetics would allow detecting subgroups of individuals having different patterns of preferences in relation to variable(s) of interest, e.g., complexity, symmetry, color, etc. After detecting these subgroups, shared characteristics (e.g., personality traits, mood, cultural background, art education, etc.) of the individuals belonging to particular subgroups can be further investigated to increase our understanding of the mechanisms underlying the aesthetic preferences.

In this study, adopting an exploratory and assumption-free approach rather than a hypothesis driven one, we aimed to demonstrate the existence of differences in complexity preferences of people, and argue against a universal rule of inverted U-curve for explaining the complexity-liking relationship. Concretely, using abstract computer-generated art as stimuli, we first show that liking versus complexity ratings averaged across all participants result in an inverted U-curve as often found in the literature. Next, by clustering participants based on their liking rating patterns, we demonstrate that this inverted U-curve is a result of combining two different liking versus complexity functions from two groups of participants. Finally, we show response time differences between these two groups of participants and discuss the implications of our results.

## Materials and Methods

### Participants

Thirty participants (average age ± standard deviation: 21.3 ± 3.5, 19 female) participated in the experiment in exchange for course credit or monetary compensation. They all had normal or corrected to normal vision. The study was approved by the Radboud University Ethics Committee Faculty of Social Sciences, and all participants provided written informed consent in accordance with the Declaration of Helsinki.

### Stimuli

As stimuli, we generated statistical geometric patterns (SGPs) using a space filling algorithm that randomly places non-overlapping geometric shapes that monotonically decrease in size (Shier, 2011; Shier and Bourke, 2013). The algorithm used in the present study was originally developed for generating art using computer programs by Shier (2011). For various examples of his ‘algorithmic art,’ the reader is referred to Shier’s website at http://john-art.com.

**FIGURE 1:**
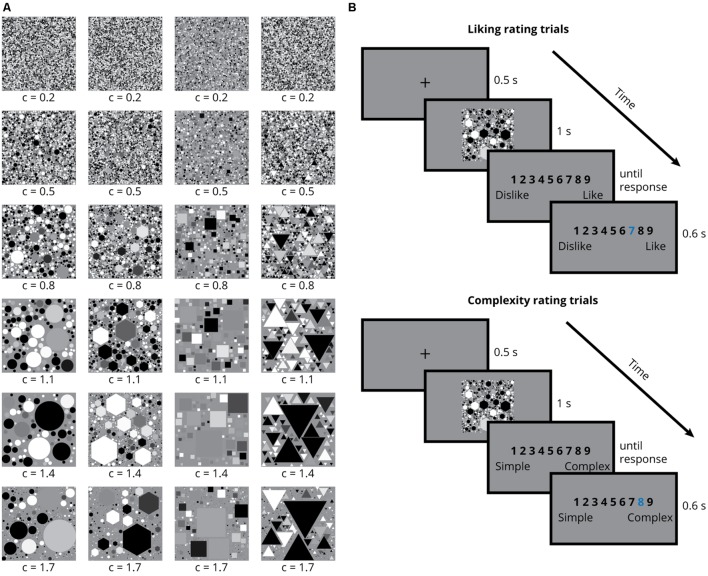
**Example stimuli and experiment design. (A)** Example stimuli with shape elements circle, hexagon, square, and triangle **(B)** Timeline of the liking and complexity rating trials.

The stimulus set^1^ consisted of 144 grayscale SGP images of basic geometric shapes; namely circle, hexagon, square, and triangle (see **Figure 1A** for a subset of the stimulus set, and supplementary materials for the complete set of stimuli used in the experiment). Each SGP image was built up by filling a square surface with same-shaped elements. The value of a parameter called ‘c’ determined both the size of the first shape element and the speed with which this size decreased (note that the parameter ‘c’ is a variable used in the generation of the stimuli, but not a measure of complexity *per se*. Further on we will relate ‘c’ to perceptual complexity). The square was filled up with element shapes until 55% of its surface area was filled, or until 5000 element shapes had been placed. The elements did not overlap. The filled-up surface had a mid-level gray color, and each element had a random gray level. We generated 36 stimuli per geometric shape with equally spaced c-values ranging from 0.2 to 1.7. Stimuli were saved as and presented in Portable Network Graphics (PNGs) file format.

### Procedure and Design

The experiment started with a short training session where participants gave nine-point Likert scale liking ratings for images presented on a computer screen. Participants were informed that the lowest rating on the scale (one) meant ‘I do not like it at all’ and the highest rating on the scale (nine) meant ‘I like it a lot,’ whereas a rating of five was neutral, and similarly for complexity ratings one meant ‘very simple’ and nine meant ‘very complex.’ No further definitions of liking or complexity were provided. Participants were encouraged to try and use the whole scale for their ratings. Through the training session, participants became familiar with the task and the type of stimulus to expect during the rest of the experiment. The stimuli presented during the training session were not used during the main rating sessions. The rest of the experiment consisted of two rating sessions: liking and complexity (**Figure 1B**). At the beginning of each session, an instruction screen reminded participants either to give ratings for how much they like the images or how complex they find the images. Every trial started with a fixation cross in the middle of a mid-level gray screen, which remained there for 500 ms, followed by the stimulus presentation for 1 s. This presentation duration is in line with the recent work by Marin and Leder (2016), who showed that presentation durations of 1 and 5 s result in very similar complexity and pleasantness ratings. After the stimulus presentation a rating screen came up, displaying a scale of numbers from one to nine. The rating screen stayed on until the response by the participant. Participant responses were entered through number keys on a computer keyboard. Once the evaluation was made by the participant, the selected number was highlighted for 600 ms before the next trial started. Each rating session consisted of 144 trials in which participants gave ratings to all stimuli. Each stimulus was presented only once per session and the stimulus presentation order was randomized for each participant. In order to avoid possible differential effects of familiarity on the liking results, liking sessions always took place before the complexity sessions.

### Analysis

#### Clustering of Participants Based on their Liking Ratings

In this study, the k-means clustering algorithm (Lloyd, 1982) was used to cluster the participants into several groups (ranging from 2 to 8) based on their liking ratings averaged across stimuli having the same c-value. Cluster analysis groups individual elements in a set in such a way that the similarity of elements assigned to the same group is maximized, whereas the similarity between different groups is minimized. Most often, similarity is defined in terms of a distance measure between the elements. K-means clustering algorithm is the simplest and most used clustering algorithm (Rokach, 2010). In this algorithm each cluster is represented by its center, which is calculated as the mean of all data points in that cluster. Starting with an initial set of cluster centers, the algorithm iteratively partitions data into *k* clusters by assigning each data point to a cluster such that the within cluster sum of squares is minimized. This is equivalent to assigning each data point to the nearest cluster.

We implemented the k-means clustering algorithm to cluster participants based on their liking ratings using the *kmeans* function of MATLAB with squared Euclidean distance measure. Cluster centers were initialized with the *k-means++* algorithm (Arthur and Vassilvitskii, 2007) as implemented in MATLAB.

In order to determine the optimum number of clusters, the average silhouette values across all data were calculated for each value of *k*, i.e., for *k* = 2, 3, 4, 5, 6, 7, and 8. The silhouette value measures a data point’s within cluster similarity in comparison to its between cluster similarity (Rousseeuw, 1987). A large average silhouette value implies a better clustering of the data, i.e., it implies that the distances between the participants in the same cluster were minimized while the separation between different clusters was maximized. Silhouette values were calculated with squared Euclidean distance measure using the *silhouette* function of MATLAB.

## Results

**FIGURE 2:**
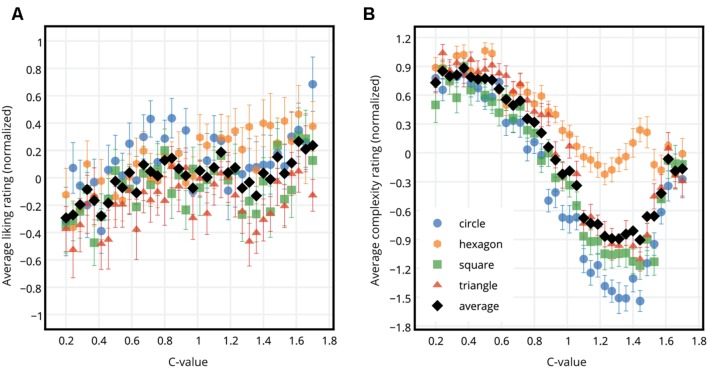
**Liking and complexity ratings for each shape type versus the c-values. (A)** Normalized liking ratings for circle, hexagon, square, and triangle SGP stimuli averaged across participants (colored dots), and across participants and shapes (black dots) versus the c-values. **(B)** Normalized complexity ratings for circle, hexagon, square, and triangle SGP stimuli averaged across participants (colored dots), and across participants and shapes (black dots) versus the c-values. Error bars show standard error of mean.

First, the liking ratings were normalized, i.e., the z-score of each rating per participant was calculated. Z-scoring brings the ratings of the individual participants to the same scale, while preserving the relative distances between individual ratings and the shape of the data (Glenberg and Andrzejewski, 2007; Mitsa, 2010). This scaling in turn allows a comparison of normalized ratings by eliminating possible confounds in the data that stem from the rating style of the participants. An example of confounds that z-scoring helps eliminate is the differences between the participants in terms of their use of the range of the rating scale (some confine their ratings to the middle of the range whereas some use the entire range). Another example is the response biases (a general tendency to give high or low ratings) of the participants. **Figure 2A** shows the normalized liking ratings for each circle, hexagon, square, and triangle SGP stimuli averaged across all participants versus the c-values of the stimuli. The liking ratings and the c-values were significantly correlated for all shapes (Pearson product-moment correlation coefficient *r_circle_* = 0.481, *p_circle_* = 0.003; *r_hexagon_* = 0.839, *p_hexagon_* < 0.001; *r_square_* = 0.481, *p_square_* = 0.003; *r_triangle_* = 0.424, *p_triangle_* = 0.010). Similarly, **Figure 2B** shows the normalized complexity ratings for each circle, hexagon, square, and triangle SGP stimuli averaged across all participants versus the c-values of the stimuli. Overall, as the c-value increased, the complexity ratings decreased (Pearson product-moment correlation coefficient *r_circle_* = -0.810, *p_circle_* < 0.001; *r_hexagon_* = -0.900, *p_hexagon_* < 0.001; *r_square_* = -0.815, *p_square_* < 0.001; *r_triangle_* = -0.850, *p_triangle_* < 0.001), however, a non-linear relationship between the complexity ratings and the c-values was visible for all shapes. In the further analysis, the main focus will be on measures of complexity (subjective and objective) and liking, but not the c-values.

Along with a subjective measure of complexity, i.e., the complexity ratings, Kolmogorov complexity approximated by the compressed file sizes of the images was used as an objective measure of stimulus complexity (Donderi and McFadden, 2005; Donderi, 2006). Kolmogorov complexity is defined as the length of the shortest program that can describe an output (Solomonoff, 1986). Kolmogorov complexity approximated by the compressed file size has been previously utilized in several other studies (Donderi and McFadden, 2005; Forsythe et al., 2008, 2011; Marin and Leder, 2013). In this study, PNG compression, which is a data format supporting lossless data compression was used. Furthermore, for comparison purposes correlational results of zip compression, which is often used in literature (Forsythe et al., 2011; Landwehr et al., 2011; Marin and Leder, 2013), are also presented (**Table 1**). The PNG compression and the zip compression performed very similarly, and were almost perfectly correlated (Pearson product-moment correlation coefficient *r* = 0.999).

**Table 1:** Bivariate correlations between various complexity measures, liking ratings, and the c-values.

Measure	Normalized complexity ratings	File size (PNG)	File size (Zip)	C-value	Normalized liking ratings
Normalized complexity ratings	1	0.946ˆ**	0.940ˆ**	-0.855ˆ**	-0.461ˆ*
File size (PNG)	0.946ˆ**	1	0.999ˆ**	-0.863ˆ**	-0.615ˆ**
File size (Zip)	0.940ˆ**	0.999ˆ**	1	-0.865ˆ**	-0.628ˆ**
C-value	-0.855ˆ**	-0.863ˆ**	-0.865ˆ**	1	0.705ˆ**
Normalized liking ratings	-0.461ˆ*	-0.615ˆ**	-0.628ˆ**	0.705ˆ**	1


*Reported values are ox*r*-values, i.e., Pearson product-moment correlation coefficients.^∗^ indicates *p*-values less than 0.01, and ^∗∗^ indicates *p*-values less than 0.001.*

**FIGURE 3:**
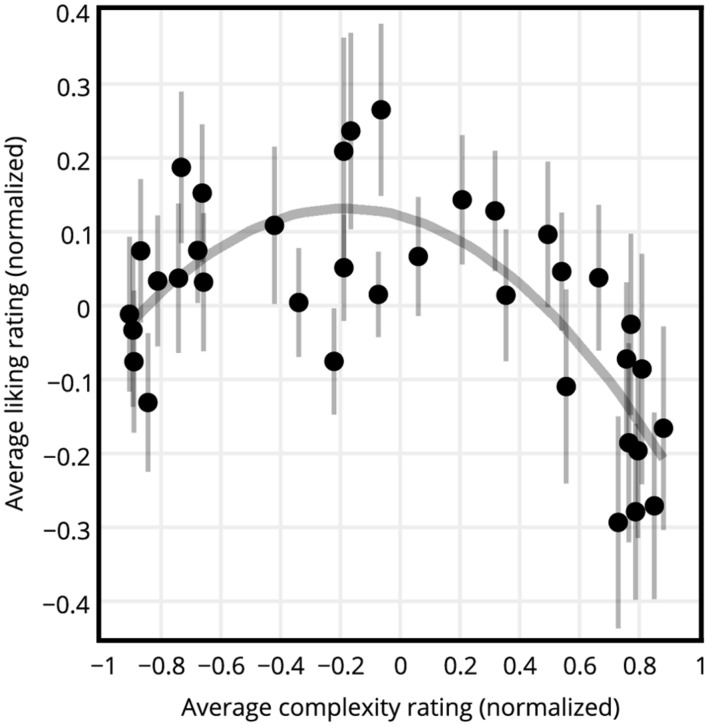
**Liking versus complexity ratings of all participants.** All ratings are normalized averages across all shapes and participants. Dots represent the stimuli, and the line represents a quadratic function fit. Error bars show standard error of mean. An inverted U-curve relationship between liking and complexity is visible.

**FIGURE 4:**
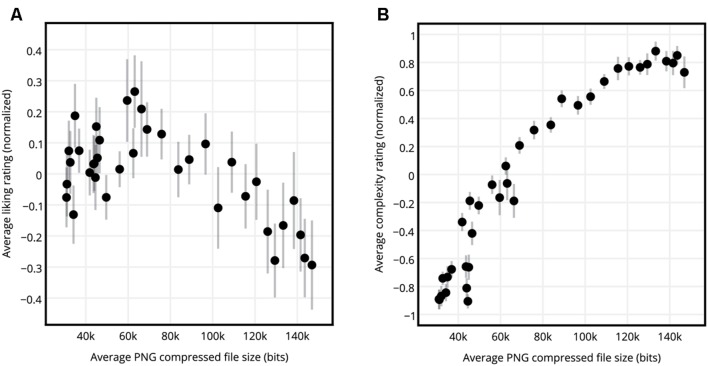
**Liking and complexity ratings versus the PNG compressed file sizes. (A)** Normalized average liking ratings for circle, hexagon, square, and triangle SGP stimuli averaged across shapes and participants, versus the average PNG compressed file sizes of the stimuli. **(B)** Normalized average complexity ratings for circle, hexagon, square, and triangle SGP stimuli averaged across shapes and participants, versus the average PNG compressed file sizes of the stimuli. Error bars show standard error of mean.

For each participant the normalized liking ratings of circle, hexagon, square, and triangle SGP stimuli having the same c-value were averaged, resulting in 36 liking ratings per participant. The same procedure was repeated for the complexity ratings, resulting in 36 complexity ratings per participant. **Figure 3** shows the normalized liking versus complexity ratings averaged across participants. The graph of liking ratings versus the complexity ratings revealed an inverted U-curve. **Figure 4** shows the normalized liking and complexity ratings for each stimulus averaged across all shape types and participants versus the PNG compressed file sizes of the stimuli averaged across all shape types. Similar to the relationship between liking and subjective complexity ratings presented in **Figure 3**, liking ratings and the PNG compressed file sizes displayed an inverted U-curve relationship (**Figure 4A**). Furthermore, the average liking ratings and the file sizes were significantly negatively correlated (see **Figure 4A** and **Table 1**). On the other hand, as expected, complexity ratings increased with increased file size (**Figure 4B**). The two measures of complexity (i.e., the subjective ratings and the objective file sizes) were significantly and highly correlated (**Table 1**).

Next, a clustering approach was adopted in order to investigate the role of individual differences in forming this relationship by identifying subgroups of individuals with different complexity preferences. As described earlier, normalized liking ratings of circle, hexagon, square, and triangle SGP stimuli having the same c-value were averaged, resulting in 36 liking ratings per participant. These liking ratings formed the input to the clustering algorithm. That is, the input to the clustering algorithm was a 30 by 36 matrix, containing the 36 normalized liking ratings for each one of the 30 participants. Using k-means clustering algorithm, several groupings of participants (*k* = 2, 3, 4, 5, 6, 7, 8) were identified. Specifically, participants were clustered into 2, 3, 4, 5, 6, 7, and 8 different subgroups based on their liking ratings averaged across stimuli having the same c-value. Afterward, to determine the optimal number of clusters among the formed subgroups, average silhouette values of the identified clusters were calculated.

**FIGURE 5:**
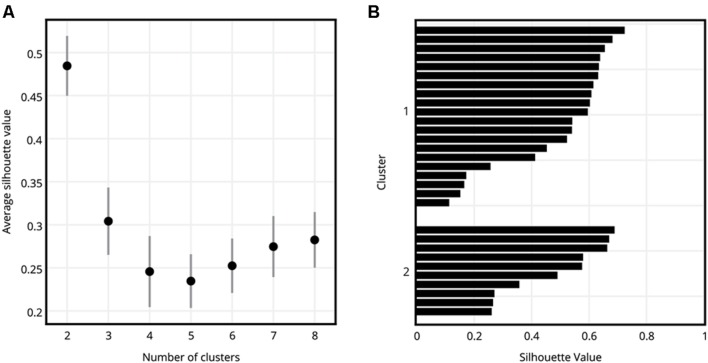
**Evaluation of grouping of participants into different numbers of clusters. (A)** Average silhouette values of clusters for *k* = 2, 3, 4, 5, 6, 7, and 8. Error bars show standard error of mean. *k* = 2, where the participants were divided into two clusters, had a significantly higher average silhouette value than the remaining numbers of clusters. **(B)** Silhouette value distribution of participants as they were assigned to Cluster 1 and Cluster 2, where *k* = 2.

Comparison of average silhouette values showed that the clusters with *k* = 2 had a significantly larger average silhouette value (**Figure 5A**) than all other values of *k* (Student’s *t*-test, *p* < 0.05 for all comparisons, Bonferroni corrected for multiple comparisons), meaning that for this dataset, the most appropriate grouping of the participants could be obtained when they were clustered into just two groups (Rousseeuw, 1987). Therefore, further analyses were performed only on the clusters obtained with *k* = 2. **Figure 5B** shows the silhouette values of participants as they were assigned to Cluster 1 and Cluster 2. Cluster 1 consisted of 20 participants (average age 25.1 ± 3.3, 7 males and 13 females), and Cluster 2 consisted of 10 participants (average age 22.7 ± 3.5, four males and six females).

**FIGURE 6:**
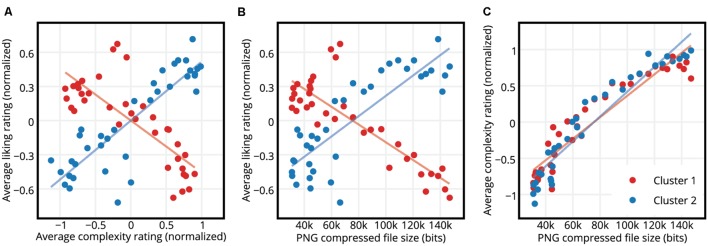
**Ratings of the participants in the two clusters. (A)** Normalized average liking versus complexity ratings of participants in Clusters 1 and 2. While participants in Cluster 1 liked the stimuli more as the stimulus complexity decreased, participants in Cluster 2 liked the more complex stimuli more. The dots represent the stimuli and the lines represent the regression lines. **(B)** Normalized average liking ratings of participants in Clusters 1 and 2 versus Kolmogorov complexity approximated by PNG compressed file sizes of the stimuli. The opposite patterns of the two clusters observed for liking versus subjective complexity in panel A are also observed here for liking versus Kolmogorov complexity. **(C)** Normalized average complexity ratings of the participants in Cluster 1 and Cluster 2 versus Kolmogorov complexity approximated by PNG compressed file sizes of the stimuli. There are no discernible differences between the average complexity ratings of the participants in different clusters.

Plotting the average liking versus complexity ratings of these two clusters separately revealed a negative relationship between liking and complexity ratings for Cluster 1 (Pearson product-moment correlation coefficient *r* = -0.789, *p* < 0.001), and a positive relationship between liking and complexity ratings for Cluster 2 (Pearson product-moment correlation coefficient *r* = 0.874, *p* < 0.001). See **Figure 6A** for the results. In other words, participants in Cluster 1 liked the simple stimulus images more than the complex ones and participants in Cluster 2 liked the complex stimulus images more than the simple ones.

The same patterns were found for liking versus the PNG compressed file sizes (**Figure 6B**). Concretely, average liking ratings of participants in Cluster 1 and PNG compressed file sizes of the stimuli were highly negatively correlated (Pearson product-moment correlation coefficient *r* = -0.849, *p* < 0.001), whereas average liking ratings of participants in Cluster 2 and PNG compressed file sizes of the stimuli were highly positively correlated (Pearson product-moment correlation coefficient *r* = 0.842, *p* < 0.001). On the other hand, average complexity ratings of the participants in the two clusters were very similar (**Figure 6C**). The average complexity rating vs. the PNG compressed file size regression slopes of the two clusters did not differ significantly [ANCOVA, *F*(1,68) = 3.07, *p* = 0.085], suggesting that the perceived complexities of the stimuli did not differ between the two clusters.

Additionally, in order to statistically compare whether a single inverted U-curve pattern (i.e., a quadratic function) or a combination of two different preference patterns belonging to the two clusters of participants (i.e., combination of two linear functions) better explain the liking-complexity relationship in our data, we performed regression analyses. Specifically we compared the following two generalized linear mixed models:

$$Liking=\beta_{0}+\beta_{1}Complexity+\beta_{2}{Complexity}^{2}+b_{0}Participant+\epsilon$$

$$Liking=\beta_{0}+\beta_{1}Complexity+\beta_{2}Cluster+\beta_{3}Complexity\times Cluster+b_{0}Participant+\epsilon$$

Where *β_i_* denotes the fixed-effect coefficients, *b_0_* denotes the random-effect coefficients and *ε* denotes the residuals. Random-effects terms were included in both models in order to account for the repeated measures structure of the data. Models were implemented using MATLAB. Since liking ratings were observed to be normally distributed, normally distributed responses and identity link function options were selected during the implementation. **Table 2** shows the estimated coefficients of the two models.

**Table 2:** Estimated fixed-effect coefficients of the quadratic and cluster-based models.

Model	Coefficient name	Estimate	*SE*	DF	*t*-statistic	*p*-value	Lower CI (95%)	Upper CI (95%)
Fixed-effect coefficients of the quadratic model	Intercept	0.067	0.026	1077	2.530	0.012	0.015	0.119
	Complexity	-0.045	0.024	1077	-1.912	0.056	-0.092	0.001
	Complexity^2^	-0.117	0.034	1077	-3.425	<0.001	-0.185	-0.050

Fixed-effect coefficients of the cluster-based model	Intercept	~0	0.048	1076	~0	1	-0.095	0.095
	Complexity	-1.004	0.064	1076	-15.662	<0.001	-1.130	-0.878
	Cluster	~0	0.034	1076	~0	1	-0.067	0.067
	Complexity ^∗^ Cluster	0.719	0.045	1076	15.978	<0.001	0.631	0.807


*The meanings of the abbreviations in the column headers are as follows: SE, standard error; DF, degrees of freedom; CI, confidence interval.*

Next, the two models were compared using a simulated likelihood ratio test with 1000 simulations. **Table 3** shows the results of simulated likelihood ratio test. The cluster-based model had lower Akaike information criterion (AIC) and Bayesian information criterion (BIC) values than the quadratic model, indicating that the cluster-based model is the better fitting model (Hox, 2002). Note that the *p*-value for the simulated likelihood ratio test was less than 0.001, further demonstrating that the cluster based model significantly better explains the data.

**Table 3:** Simulated likelihood ratio test results.

Model	DF	AIC	BIC	Log likelihood	LRT-statistic	*p*-value (95% CIs)
Quadratic model	5	1923.5	1948.5	-956.77	217.49	<0.001 (0.00002–0.006)
Cluster-based model	6	1708	1737.9	-848.02		


*The meanings of the abbreviations in the column headers are as follows: DF, degrees of freedom (number of free parameters in the model); AIC, Akaike information criterion for the model; BIC, Bayesian information criterion for the model; Log likelihood, maximized log likelihood for the model; LRT-statistic, likelihood ratio test statistic; CI, confidence interval.*

As a final analysis, we looked at the response times of the participants. Participants in Cluster 1, who had a preference toward simpler patterns, had significantly shorter average response times (*M* = 1.639 s, *SD* = 0.108 s) for liking ratings than participants in Cluster 2 (*M* = 1.803 s, *SD* = 0.197), who preferred more complex patterns (Student’s *t*-test, *p* < 0.001).The average response times for complexity ratings of participants in Cluster 1 (*M* = 1.477 s, *SD* = 0.103 s) and Cluster 2 (*M* = 1.494 s, *SD* = 0.210) did not differ (Student’s *t*-test, *p* = 0.396).

## Discussion

In this study, abstract computer-generated art of varying levels of complexity was evaluated in terms of liking and complexity. Consistent with the literature, we found an inverted U-curve relationship between liking and complexity of these images. Next, utilizing a data-driven clustering approach, we revealed subgroups of people with different preferences for image complexity. Note that we neither had an *a priori* assumption regarding the number of clusters nor the shape of the liking-complexity relationships in the different clusters. Following the clustering approach, two subgroups having opposite preferences for image complexity (one which liked simple patterns more than the complex ones, and another which liked complex patterns more than the simple ones) were identified. The two groups differed in terms of how much they liked the complex or simple patterns, but not how they perceived complexity. Furthermore, a comparison of a quadratic model (representing the inverted U-curve relationship) and a cluster-based model (representing the combination of two linear relationships) revealed that the cluster-based model was a better fit to the data at hand. Interestingly, the group of participants who liked the simpler patterns more were faster in their liking evaluations compared to the group that preferred complex patterns. In contrast, there were no differences between the groups in terms of the time they took to evaluate the complexity of the images.

### Possible Interpretations of the Results

An important point to keep in mind when attempting to interpret our results is the exploratory – rather than hypothesis driven – approach that we took in this study. One implication of this approach is that we can describe the differences between the two identified clusters in terms of the measurements at hand, but cannot make claims about causal relationships regarding the factors influencing these results. For example, by clustering participants based on their liking ratings, a non-random assignment of participants to one of the two groups was introduced. This in turn might have increased the possibility of groups having different distributions in terms of several unidentified factors such as intelligence, personality, art experience, motivation, etc. Because one or multiple of these unidentified factors might have had an influence on the response time results, the strength of conclusions that can be derived from the results about the response time differences becomes limited. In other words, our interpretations of the results should be viewed in light of the fact that all the analyses and statements trying to characterize the clusters are *post hoc*.

We showed that the group of participants who preferred simpler patterns were faster in their liking evaluations compared to the group that preferred complex patterns. The liking rating response time differences that we identified between the groups were not present for the complexity rating responses. This leads us to think that the unidentified factors contributing to these response time differences should be more specifically related to aesthetic evaluation of images (e.g., art experience/education, art interest, motivation, personality), rather than a more general factor which would also effect the complexity evaluation response times (e.g., intelligence). Furthermore, on average, the complexity of the images were perceived similarly in the two clusters as shown in **Figure 6C.** This suggests that the preference differences of the two clusters cannot be explained by the differences in their perception of complexity.

A partial explanation to our results could be provided by the fluency theory (Reber et al., 2004). According to the fluency theory, experience of fluent processing results in positive affect toward stimuli. Based on this theory, one would expect decreased liking toward complex (and hence less fluently processed) stimuli compared to simpler (and hence more fluently processed) stimuli. The majority of the participants in our experiment (20 out of 30) were assigned to Cluster 1 which showed a monotonic decrease in liking for stimuli with increased complexity. Their behavior is in line with the fluency theory. However, the average tendency of the remaining ten participants in Cluster 2 was a monotonic increase in liking for stimuli with increased complexity, which is difficult to account for with the fluency theory of aesthetic liking. Future studies can investigate differential effects of fluency on participants who have different complexity preferences in order to evaluate the merit of such an interpretation.

A recent framework by Graf and Landwehr (2015), called the Pleasure-Interest Model of Aaesthetic Liking (PIA Model), claims to provide a better explanation for contradictory preference patterns for aesthetic stimuli that are easy or difficult to process. According to Graf and Landwehr (2015), an aesthetic object may be processed in two stages. First an automatic processing takes place, and then if the viewer is motivated enough to process the stimuli further, a controlled processing follows. Similar to the fluency theory, the PIA Model predicts that merely automatic processing of stimuli would result in a monotonic decrease of liking as the stimulus complexity increases. The model further predicts that controlled processing could result in an inverted U-curve if the complexity levels of stimuli are high enough to cause dislike and confusion. Our results do not conflict with this model, however to explain the results in terms of the PIA model, assumptions need to be made. These assumption are related to the motivation levels of the participants, perceived complexity of the stimulus material and factors affecting the response times. Testing these assumptions would go beyond the scope of this paper. Additional studies would be required to test the PIA model.

### Relation to Past and Future Research

We believe that it is important to try and characterize the groups of people with different complexity preferences as found in the present study. Previous studies give some valuable insight in this direction. For example, preference for complexity has long been associated with creativity and artistic tendencies (Barron and Welsh, 1952; Mackinnon, 1962; Barron, 1963; McWhinnie, 1968). In fact, the Barron-Welsh art scale (Barron and Welsh, 1952) which measures an individual’s preference for complexity has been used to assess creativity in several previous studies (for a review, see Gough et al., 1996). Along with creativity and artistic tendencies, a person’s age has been shown to affect their preference for complexity. Particularly, older individuals have been shown to prefer simpler visual stimuli (Munsinger et al., 1964; Alpaugh and Birren, 1977; Crosson and Robertson-Tchabo, 1983). In the present study, although the average age of Cluster 1 (*M* = 25.1, *SD* = 3.3) was higher than that of Cluster 2 (*M* = 22.7, *SD* = 3.5), this difference was very small and not significant (Student’s *t*-test, *p* = 0.082). However, since our sample of participants consisted of a similarly aged group of young adults between the ages of 20 and 32, such a result was expected.

More recently, the personality traits ‘openness to experience’ and ‘conscientiousness’ (of the Big Five personality inventory by Goldberg, 1999) along with ‘frequency of visits to galleries/museums’ have been shown to correlate with preference for more complex art (Chamorro-Premuzic et al., 2010; Cleridou and Furnham, 2014). However, it is important to note that these correlations were only able to account for a limited amount of the variability in the data. Nevertheless, it is important for future studies to investigate the distribution of personality traits as well as artistic and creative tendencies within the clusters of participants who have different complexity preferences.

Here, we focused on complexity because it is a relatively general and overarching concept that has been shown to relate to the appreciation of a stimulus on several dimensions such as the number of elements, irregularity of shape and arrangement, heterogeneity of elements and asymmetry (Berlyne, 1971). Additionally, in the past decades various measures of pattern complexity have been suggested, developed and shown to be relevant for perception, e.g., Birkhoff’s complexity elements, *C* (Birkhoff, 1933; Eysenck, 1941), the amount of structural information (Leeuwenberg, 1969, 1971; Boselie, 1984; van der Helm et al., 1992), amount of algorithmic information, i.e., Kolmogorov complexity (Donderi and McFadden, 2005; Marin and Leder, 2013), and the amount of self-similarity, i.e., fractal dimension (Mandelbrot, 1977, 1981; Cutting and Garvin, 1987; Spehar et al., 2003). Therefore, we found complexity to be a good starting point for studying the individual differences in preferences. It would however be interesting to further investigate how the individual differences in complexity preferences relate to the previously identified individual differences in preferences for other visual perceptual attributes, such as harmony (Palmer and Griscom, 2013) or symmetry (Jacobsen and Höfel, 2002).

Recently, Spehar et al. (2015) have shown that participants’ visual sensitivity to various visual properties (e.g., amplitude spectrum characteristics of synthetic images and spatial frequency of sine-wave gratings) highly correlate with their visual preferences. In a future study, it would be very interesting to investigate the relationship between visual sensitivity of individuals and their preference for complexity. Furthermore, another interesting question to investigate would be whether or not a clustering of participants as found in our study would be observed for more complex artistic stimuli with interpretable contents.

## Conclusion

The results of our study have both theoretical and practical implications. Here, we showed that one of the most well-known rules of aesthetic preference, i.e., the inverted U-curve of preference for complexity, can in fact be an artifact that arises from selecting a non-ideal analysis method. By employing an averaging approach, most experimental aesthetics studies risk reaching conclusions about a non-existent average observer. Besides the need for utilization of new analysis methods that take into account the differences between individuals, theoretical implications of this finding include a need for re-evaluation of established rules of human aesthetic preferences and revision of existing theories, in a way that would explain e.g., the monotonically increasing and monotonically decreasing liking as a function of complexity, rather than a global mid-level complexity preference. Similarly, in the practical sense, our results are relevant for designers and artists. Rather than opting for a mid-level complexity in their designs to please the average observer, they can utilize more targeted design strategies for appealing to different groups of individuals. However, characteristics of these groups remain to be identified.

## Author Contributions

YG, RJ, and RvL designed the research. YG conducted the research and analyzed the data. YG, RJ, and RvL interpreted the results and wrote the manuscript.

## Conflict of Interest Statement

The authors declare that the research was conducted in the absence of any commercial or financial relationships that could be construed as a potential conflict of interest.

## Footnotes

1 Stimulus material and data are available upon email request from the corresponding author.

## References

[B1] Aks D. J. Sprott J. C. (1996). Quantifying aesthetic preference for chaotic patterns. *Empir. Stud. Arts* 14 1–16. 10.2190/6V31-7M9R-T9L5-CDG9

[B2] Alpaugh P. K. Birren J. E. (1977). Variables affecting creative contributions across the adult life span. *Hum. Dev.* 20 240–248. 10.1159/000271559 903112

[B3] Arthur D. Vassilvitskii S. (2007). “K-means++: the advantages of careful seeding,” in *Proceedings of the Eighteenth Annual ACM-SIAM Symposium on Discrete Algorithms*, New Orleans, LA, 1027–1035.

[B4] Barron F. (1963). *Creativity and Psychological Health: Origins of Personal Vitality and Creative Freedom.* Princeton, NJ: Van Nostrand.

[B5] Barron F. Welsh G. S. (1952). Artistic perception as a possible factor in personality style: its measurement by a figure preference test. *J. Psychol.* 33 199–203. 10.1080/00223980.1952.9712830

[B6] Berlyne D. E. (1963). Complexity and incongruity variables as determinants of exploratory choice and evaluative ratings. *Can. J. Psychol.* 17 274–290. 10.1037/h0092883 14048839

[B7] Berlyne D. E. (1971). *Aesthetics and Psychobiology.* New York, NY: Appleton-Century-Crofts.

[B8] Berlyne D. E. (1977). Psychological aesthetics, speculative and scientific. *Leonardo* 10 56–58. 10.2307/1573634

[B9] Berlyne D. E. Ogilvie J. C. Parham L. C. (1968). The dimensionality of visual complexity, interestingness, and pleasingness. *Can. J. Psychol.* 22 376–387. 10.1037/h0082777 5724480

[B10] Birkhoff G. D. (1933). *Aesthetic Measure.* Cambridge, MA: Harvard University Press.

[B11] Boselie F. (1984). Complex and simple proportions and the aesthetic attractivity of visual patterns. *Perception* 13 91–96. 10.1068/p130091 6504684

[B12] Chamorro-Premuzic T. Burke C. Hsu A. Swami V. (2010). Personality predictors of artistic preferences as a function of the emotional valence and perceived complexity of paintings. *Psychol. Aesthet. Creat. Arts* 4 196–204. 10.1037/a0019211

[B13] Chamorro-Premuzic T. Reimers S. Hsu A. Ahmetoglu G. (2009). Who art thou? Personality predictors of artistic preferences in a large UK sample: the importance of openness. *Br. J. Psychol.* 100(Pt 3), 501–516. 10.1348/000712608X366867 19026107

[B14] Clatworthy J. Buick D. Hankins M. Weinman J. Horne R. (2005). The use and reporting of cluster analysis in health psychology: a review. *Br. J. Health Psychol.* 10 329–358. 10.1348/135910705X25697 16238852

[B15] Cleridou K. Furnham A. (2014). Personality correlates of aesthetic preferences for art, architecture, and music. *Empir. Stud. Arts* 32 231–255. 10.2190/EM.32.2.f

[B16] Crosson C. W. Robertson-Tchabo E. A. (1983). Age and preference for complexity among manifestly creative women. *Hum. Dev.* 26 149–155. 10.1159/000272878

[B17] Cutting J. E. Garvin J. J. (1987). Fractal curves and complexity. *Percept. Psychophys.* 42 365–370. 10.3758/BF03203093 3684493

[B18] Donderi D. C. (2006). Visual complexity: a review. *Psychol. Bull.* 132 73–97. 10.1037/0033-2909.132.1.73 16435958

[B19] Donderi D. C. McFadden S. (2005). Compressed file length predicts search time and errors on visual displays. *Displays* 26 71–78. 10.1016/j.displa.2005.02.002

[B20] Eysenck H. J. (1941). The empirical determination of an aesthetic formula. *Psychol. Rev.* 48 83–92. 10.1037/h0062483

[B21] Farley F. H. Weinstock C. A. (1980). Experimental aesthetics: children’s complexity preference in original art and photoreproductions. *Bull. Psychon. Soc.* 15 194–196. 10.3758/BF03334506

[B22] Forsythe A. Mulhern G. Sawey M. (2008). Confounds in pictorial sets: the role of complexity and familiarity in basic-level picture processing. *Behav. Res. Methods* 40 116–129. 10.3758/BRM.40.1.116 18411534

[B23] Forsythe A. Nadal M. Sheehy N. Cela-Conde C. J. Sawey M. (2011). Predicting beauty: fractal dimension and visual complexity in art. *Br. J. Psychol.* 102 49–70. 10.1348/000712610X498958 21241285

[B24] Glenberg A. M. Andrzejewski M. E. (2007). *Learning from Data: An Introduction to Statistical Reasoning*, 3rd Edn New York, NY: Lawrence Erlbaum Associates, Inc.

[B25] Goldberg L. R. (1999). “A broad-bandwidth, public domain, personality inventory measuring the lower-level facets of several five-factor models,” in *Personality Psychology in Europe* Vol. 7 eds Mervielde I. Deary I. De Fruyt F. Ostendorf F. (Tilburg: Tilburg University Press), 7–28.

[B26] Gough H. G. Hall W. B. Bradley P. (1996). “Forty years of experience with the Barron-Welsh Art scale,” in *Unusual Associates: A Festschrift for Frank Barron*, eds Barron F. Montuori A. (New York, NY: Hampton Press), 252–301.

[B27] Graf L. K. M. Landwehr J. R. (2015). A dual-process perspective on fluency-based aesthetics: the pleasure-interest model of aesthetic liking. *Pers. Soc. Psychol. Rev.* 19 395–410. 10.1177/1088868315574978 25742990

[B28] Hox J. J. (2002). *Multilevel Analysis: Techniques and Applications (First).* Mahwah, NJ: Lawrence Erlbaum Associates, Inc.

[B29] Imamoglu Ç (2000). Complexity, liking and familiarity: architecture and non-architecture Turkish students’ assesments of traditional and modern house facades. *J. Environ. Psychol.* 20 5–16. 10.1006/jevp.1999.0155

[B30] Jacobsen T. (2004). Individual and group modelling of aesthetic judgment strategies. *Br. J. Psychol.* 95(Pt 1), 41–56. 10.1348/000712604322779451 15005867

[B31] Jacobsen T. Höfel L. (2002). Aesthetic judgments of novel graphic patterns: analyses of individual judgments. *Percept. Mot. Skills* 95(3 Pt 1), 755–766. 10.2466/pms.2002.95.3.755 12509172

[B32] Landwehr J. R. Labroo A. A. Herrmann A. (2011). Gut liking for the ordinary: incorporating design fluency improves automobile sales forecasts. *Mark. Sci.* 30 416–429. 10.1287/mksc.1110.0633

[B33] Leeuwenberg E. L. (1969). Quantitative specification of information in sequential patterns. *Psychol. Rev.* 76 216–220. 10.1037/h0027285 5778471

[B34] Leeuwenberg E. L. (1971). A perceptual coding language for visual and auditory patterns. *Am. J. Psychol.* 84 307–349. 10.2307/1420464 5142580

[B35] Lloyd S. (1982). Least squares quantization in PCM. *IEEE Trans. Inf. Theory* 28 129–137. 10.1109/TIT.1982.1056489

[B36] Mackinnon D. W. (1962). The nature and nurture of creative talent. *Am. Psychol.* 17 484–495. 10.1037/h0046541

[B37] Mallon B. Redies C. Hayn-Leichsenring G. U. (2014). Beauty in abstract paintings: perceptual contrast and statistical properties. *Front. Hum. Neurosci.* 8:161 10.3389/fnhum.2014.00161 PMC3968763 24711791

[B38] Mandelbrot B. B. (1977). *Fractals: Form, Chance and Dimension.* San Francisco, CA: Freeman.

[B39] Mandelbrot B. B. (1981). Scalebound or scaling shapes: a useful distinction in the visual arts and in the natural sciences. *Leonardo* 14 45–47. 10.2307/1574481

[B40] Marin M. M. Leder H. (2013). Examining complexity across domains: relating subjective and objective measures of affective environmental scenes, paintings and music. *PLoS ONE* 8:e72412 10.1371/journal.pone.0072412 PMC3745471 23977295

[B41] Marin M. M. Leder H. (2016). Effects of presentation duration on measures of complexity in affective environmental scenes and representational paintings. *Acta Psychol.* 163 38–58. 10.1016/j.actpsy.2015.10.002 26595281

[B42] McManus I. C. Cook R. Hunt A. (2010). Beyond the Golden Section and normative aesthetics: why do individuals differ so much in their aesthetic preferences for rectangles? *Psychol. Aesthet. Creat. Arts* 4 113–126. 10.1037/a0017316

[B43] McWhinnie H. J. (1968). A review of research on aesthetic measure. *Acta Psychol.* 28 363–375. 10.1016/0001-6918(68)90025-5

[B44] Mitsa T. (2010). *Temporal Data Mining.* Boca Raton, FL: Taylor and Francis Group, LLC.

[B45] Munsinger H. Kessen W. Kessen M. L. (1964). Age and uncertainty: developmental variation in preference for variability. *J. Exp. Child Psychol.* 1 1–15. 10.1016/0022-0965(64)90002-5

[B46] Nadal M. Munar E. Marty G. Cela-conde C. J. (2010). Visual complexity and beauty appreciation: explaining the divergence of results. *Empir. Stud. Arts* 28 173–191. 10.2190/EM.28.2.d

[B47] Nusbaum E. C. Silvia P. J. (2011). Shivers and timbres: personality and the experience of chills from music. *Soc. Psychol. Personal. Sci.* 2 199–204. 10.1177/1948550610386810

[B48] Palmer S. E. Griscom W. S. (2013). Accounting for taste: individual differences in preference for harmony. *Psychon. Bull. Rev.* 20 453–461. 10.3758/s13423-012-0355-2 23242798

[B49] Reber R. Schwarz N. Winkielman P. (2004). Processing fluency and aesthetic pleasure: is beauty in the perceiver’s processing experience? *Pers. Soc. Psychol. Rev.* 8 364–382. 10.1207/s15327957pspr0804_3 15582859

[B50] Rokach L. (2010). “A survey of clustering algorithms,” in *Data Mining and Knowledge Discovery Handbook*, 2nd Edn, eds Maimon O. Rokach L. (New York, NY: Springer Science+Business Media)269–298.

[B51] Rousseeuw P. J. (1987). Silhouettes: a graphical aid to the interpretation and validation of cluster analysis. *J. Comput. Appl. Math.* 20 53–65. 10.1016/0377-0427(87)90125-7

[B52] Saklofske D. H. (1975). Aesthetic complexity and exploratory behavior. *Percept. Mot. Skills* 41 363–368. 10.2466/pms.1975.41.2.363 1187292

[B53] Shier J. (2011). “Filling space with random fractal non-overlapping simple shapes,” in *Proceedings of ISAMA 2011*, Chicago, IL, 131–140.

[B54] Shier J. Bourke P. (2013). An algorithm for random fractal filling of space. *Comput. Graph. Forum* 32 89–97. 10.1111/cgf.12163

[B55] Silvia P. J. (2005). Cognitive appraisals and interest in visual art: exploring an appraisal theory of aesthetic emotions. *Empir. Stud. Arts* 23 119–133. 10.2190/12AV-AH2P-MCEH-289E

[B56] Solomonoff R. J. (1986). “The application of algorithmic probability to problems in artificial intelligence,” in *Uncertainty in Artificial Intelligence*, eds Kanal L. N. Lemmer J. F. (North-Holland: Elsevier Science Publishers B.V),473–491.

[B57] Spehar B. Clifford C. W. G. Newell B. R. Taylor R. P. (2003). Universal aesthetic of fractals. *Comput. Graph.* 27 813–820. 10.1016/S0097-8493(03)00154-7

[B58] Spehar B. Wong S. van de Klundert S. Lui J. Clifford C. W. G. Taylor R. P. (2015). Beauty and the beholder: the role of visual sensitivity in visual preference. *Front. Hum. Neurosci.* 9:514 10.3389/fnhum.2015.00514 PMC4585069 26441611

[B59] van der Helm P. A. van Lier R. J. Leeuwenberg E. L. (1992). Serial pattern complexity: irregularity and hierarchy. *Perception* 21 517–544. 10.1068/p210517 1437468

[B60] Vitz P. C. (1966). Preference for different amounts of visual complexity. *Behav. Sci.* 11 105–114. 10.1002/bs.3830110204 5909015

[B61] Zubin J. (1938). A technique for measuring like-mindedness. *J. Abnorm. Soc. Psychol.* 33 508–516. 10.1037/h0055441

