# Supplementary material for: Liking versus Complexity: Decomposing the Inverted U-curve
Source: Front Hum Neurosci. 2016 Mar 18;10:112. doi: 10.3389/fnhum.2016.00112 (PMC4796011; doi:10.3389/fnhum.2016.00112)
Supplement: Supplementary file 1 [file Data_Sheet_1.DOCX]

***Supplementary Material***

**Liking versus complexity: Decomposing the inverted U-curve by accounting for individual differences**

**Yağmur Güçlütürk ^1^*, Richard H. A. H. Jacobs ^1^, Rob van Lier ^1^**

^1^ Donders Institute for Brain, Cognition and Behaviour, Radboud University, Nijmegen, Netherlands

*** Correspondence:** Yağmur Güçlütürk, Donders Institute for Brain, Cognition and Behaviour, Radboud University, Nijmegen, Netherlands.

y.gucluturk@donders.ru.nl

1. **Stimulus Set**

The stimulus set consisted of 144 grayscale square statistical geometric pattern (SGP) images with a side length of 800 pixels. The images were generated using the algorithm developed by Shier (2011). Specifically, there were 36 circle, 36 hexagon, 36 square and 36 triangle SGPs with equally spaced c-parameter values ranging from 0.2 to 1.7. Please see below for the resized versions of the complete set of stimulus images.


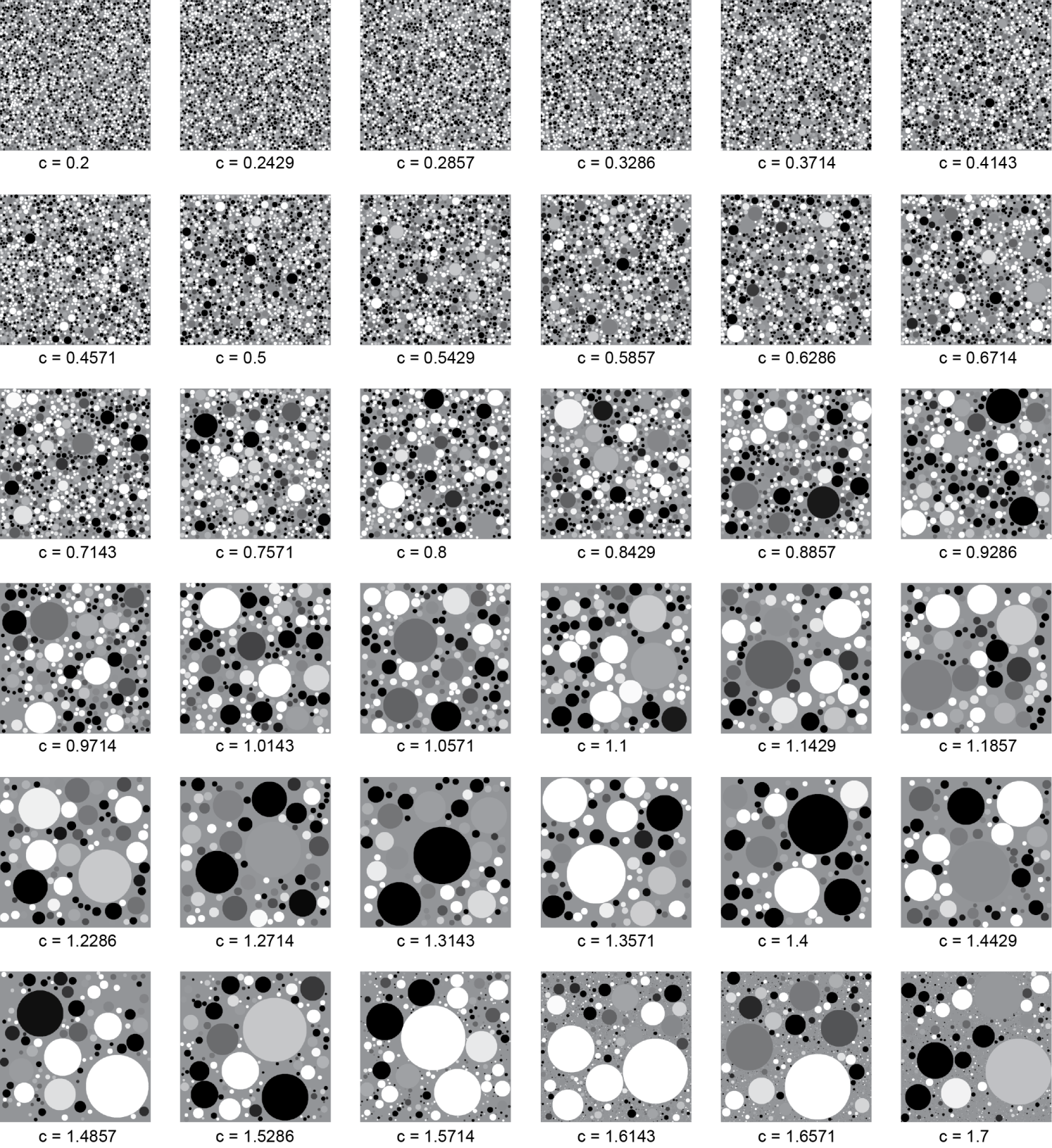


Supplementary Figure 1. Circle SGP images. Equally spaced c-parameter values range from 0.2 to 1.7.


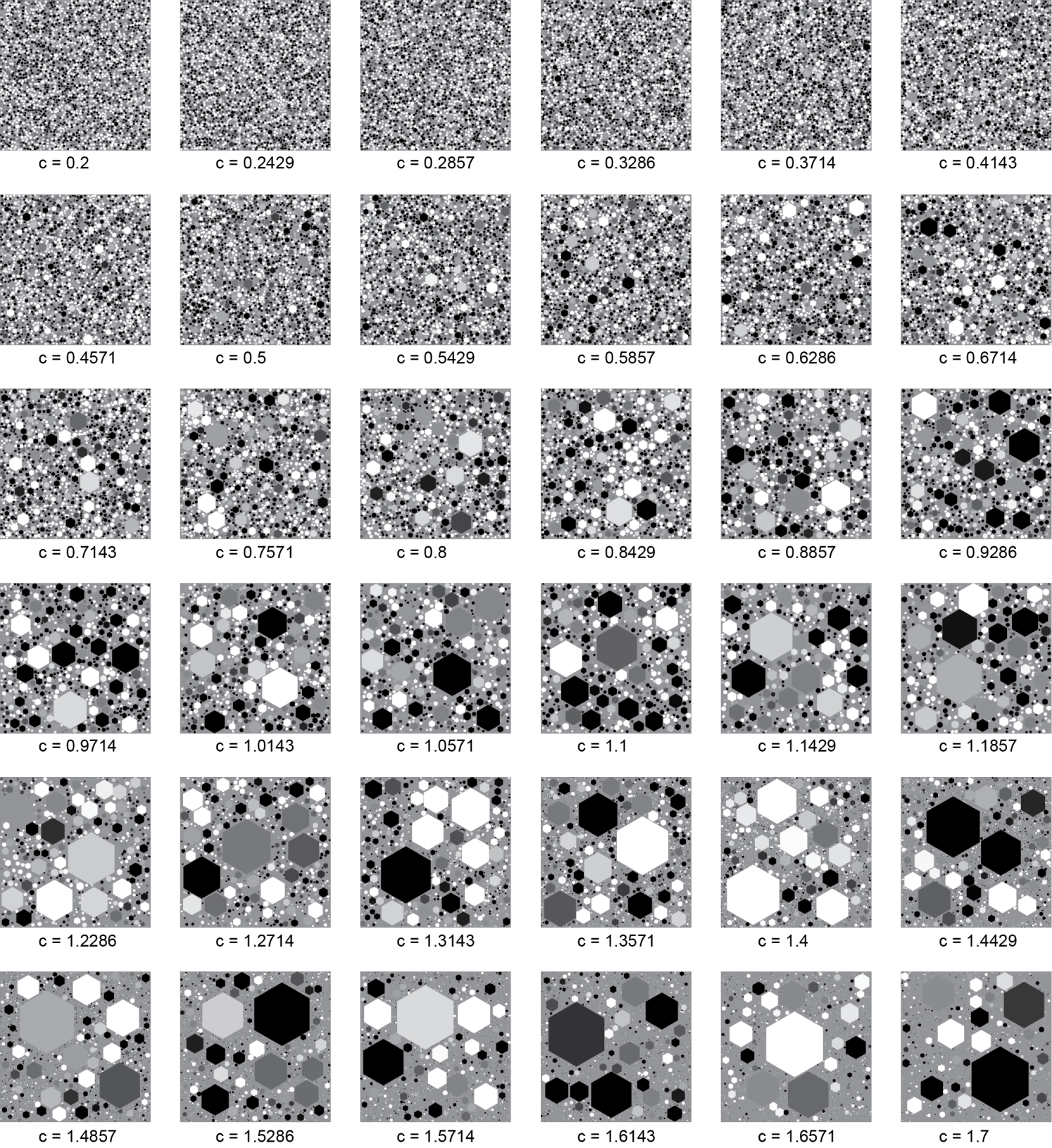


Supplementary Figure 2. Hexagon SGP images. Equally spaced c-parameter values range from 0.2 to 1.7.

**
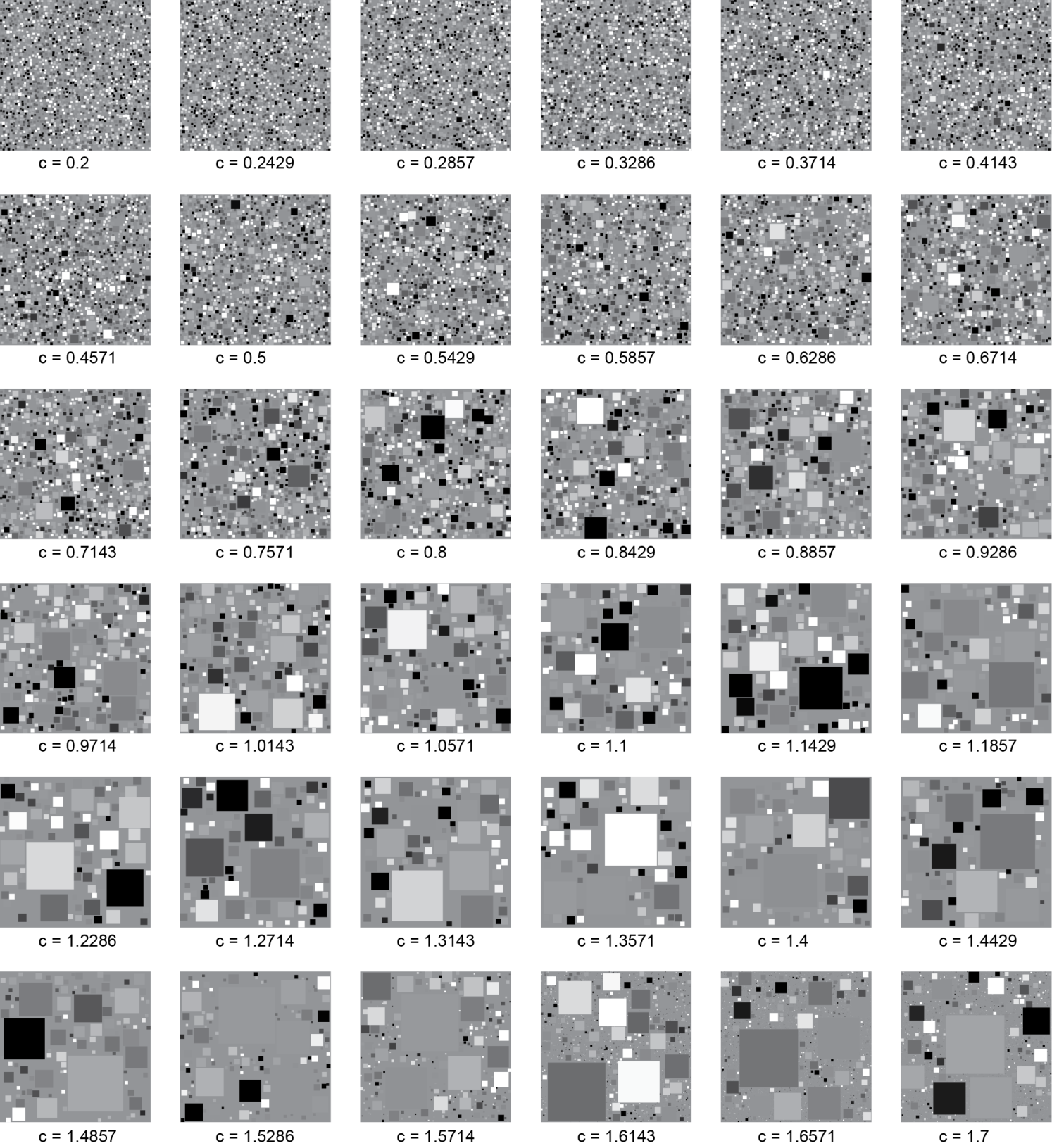
**

Supplementary Figure 3. Square SGP images. Equally spaced c-parameter values range from 0.2 to 1.7.


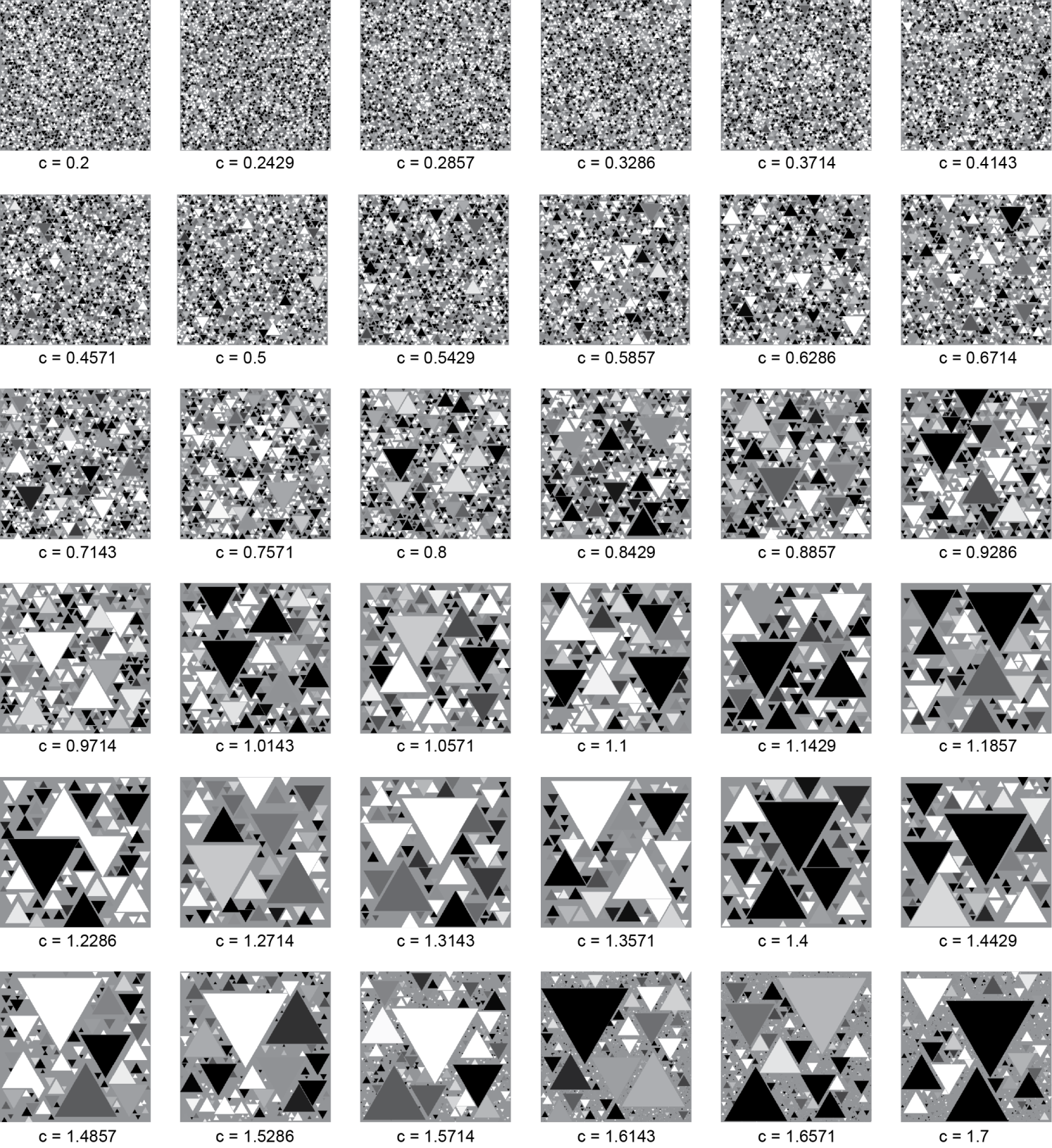


Supplementary Figure 4. Triangle SGP images. Equally spaced c-parameter values range from 0.2 to 1.7.

1. **Algorithm for Generating SGP Images**

The initial steps for generating an SGP image (as used in our stimulus set) include defining an empty enclosed surface to be filled, selecting a shape which will be used to fill this surface, defining stopping criteria and selecting a value for the algorithm parameter c. The algorithm then starts by placing the first shape at a random coordinate inside the defined enclosed surface. At the next iteration, after an intersection check ensuring that no two shapes overlap, a smaller shape is placed at another random location.

The size of the enclosed surface, the value of the algorithm parameter c, and the set maximum number of iterations together determine the size of the first shape to be placed on the surface. Furthermore, the value of parameter c also determines the speed with which the shape area decreases at each iteration. Concretely, at each iteration, the shape area decreases fast for large values of c, whereas it decreases slowly for small values of c (see Supplementary Figures 1, 2, 3 and 4).

A collection of resources regarding the algorithm for generating SGP images can be found online at John Shier’s website <http://john-art.com/stat_geom_linkpage.html>. Furthermore, Paul Bourke has a very informative website on the topic: <http://paulbourke.net/texture_colour/randomtile/>.

1. **References**

Shier, J. (2011). Filling space with random fractal non-overlapping simple shapes. In *Proceedings of ISAMA 2011* (pp. 131–140).
